# CREB and neuronal selection for memory trace

**DOI:** 10.3389/fncir.2013.00044

**Published:** 2013-03-21

**Authors:** Jieun Kim, Jeong-Tae Kwon, Hyung-Su Kim, Jin-Hee Han

**Affiliations:** Laboratory of Neural Circuit and Behavior, Department of Biological Sciences, KAIST Institute for the BioCentury, Korea Advanced Institute of Science and TechnologyDaejeon, Korea

**Keywords:** neural network, memory trace, neuronal selection, CREB, excitability, dendritic spine

## Abstract

Despite considerable progress over the past several decades, our understanding of the mechanisms underlying memory encoding, storage, and expression in a complex neural network are far from complete. In particular, how some neurons rather than others are selectively engaged to encode memory remains largely unknown. Using virus-mediated gene delivery into a small subset of neurons in a given network, molecular imaging of neuronal activity, pharmacological perturbation of specific neurons' activity and animal behavior assays, recent studies have begun to provide insight into molecular and cellular mechanisms responsible for the selection of neurons for inclusion into a memory trace. Here, we focus on a review of recent findings supporting the hypothesis that the level of the transcription factor CREB (cAMP/Ca^2+^-response element binding protein) is a key factor governing which neurons are recruited to a given memory trace. These recent findings open a new perspective on memory trace at the neural circuit level and also raise many important questions. Future studies employing more advanced neurobiological techniques for targeting defined populations of neurons and manipulating their activity in time and space in a complex neural network will give answers to these newly emerging questions and extend our understanding of the neurobiological basis of the memory trace.

A major challenge in neuroscience is to understand how memory is formed, stored, and expressed in our brain. In recent decades, the majority of research in the learning and memory field has focused on the molecular and cellular basis of memory (Kandel, 2009). These studies have identified many molecules essential for memory formation and revealed the cellular correlate—synaptic plasticity—and its underlying mechanisms (Bliss and Lømo; 1973; Bliss et al., 2007; Kandel, 2009, 2012). However, because the brain is a complex structure formed by connections of billions of nerve cells, the memory trace, which is the physical substrate of memory in the brain, must also be understood at the neural circuit level (Lashley, 1950; McGaugh, 1972; Schacter, 2001; Thompson, 2005; Neves et al., 2008; Mayford et al., 2012). Among the key unanswered questions: which specific neurons store particular memories?

Several studies have shown that memory might be stored in a sparsely distributed specific set of neurons in the brain that form a unique memory trace; accordingly, only a portion of eligible neurons are recruited into a specific memory. For instance, imaging of activity-dependent induction of the immediate-early gene Arc (activity-regulated cytoskeleton-associated protein, also known as Arg3.1) showed that a novel environment produced activation of a sparsely distributed neuronal ensemble in the hippocampus (Guzowski et al., 1999). During fear conditioning, about 70% of neurons in the lateral amygdala (LA), a critical brain site for fear memory acquisition and storage, are thought to receive sensory inputs, but only about one-quarter exhibit learning-related synaptic plasticity (Repa et al., 2001; Rumpel et al., 2005). In the latter of these two studies, electrophysiological recording of thalamo-amygdala synapses in the rat brain showed that auditory fear conditioning induced membrane insertion of GluR1 type AMPA [2-amino-3-(3-hydroxy-5-methyl-isoxazol-4-yl)propanoic acid] receptors, a marker for synaptic plasticity (e.g., LTP), into the synapses of about one-third of LA neurons. Using a carboxyl cytoplasmic tail construct that interferes with GluR1 insertion and blocks plasticity, this study also investigated how many LA neurons that undergo GluR1 trafficking are necessary for normal fear memory formation. Behavioral tests showed that, if GluR1 trafficking is blocked in ~10–20% of LA neurons undergoing plasticity, fear memory formation is impaired. These findings have raised important questions about memory encoding and storage in a complex neural circuit. For example, how are particular neurons recruited into a given memory trace? Are there predetermined stereotyped neurons for a specific memory? Alternatively, are particular neurons in a given neural network actively selected by some mechanism(s) at the time of learning? If a specific set of neurons is selected for encoding of memory during learning, are the selected neurons essential for the subsequent expression of that memory?

Recent studies have begun to address some of these questions, providing evidence suggesting that the transcription factor CREB governs the selection of neurons for inclusion into a fear memory trace. CREB was first described by Marc Montminy and L. M. Bilezikjian in 1987 (Montminy, 1997) as a cellular transcription factor that binds the cAMP-response element, leading to increased transcription of the somatostatin gene. CREB acts as a transcriptional activator only after it is phosphorylated by certain protein kinases, such as protein kinase A, mitogen-activated protein kinase, and calcium/calmodulin-dependent kinase (Montminy, 1997). A large body of evidence from genetic and pharmacological studies in a wide range of species indicates that CREB is essential for memory formation (Dash et al., 1990; Tully, 1991; Kaang et al., 1993; Bourtchuladze et al., 1994; Yin et al., 1994; Guzowski and McGaugh, 1997; Lamprecht et al., 1997; Bartsch et al., 1998; Silva et al., 1998; Kida et al., 2002; Pittenger et al., 2002; Josselyn et al., 2004; Restivo et al., 2009). Increasing CREB levels enhances some forms of long-lasting memory (Yin et al., 1995; Josselyn et al., 2001; Wallace et al., 2004; Jasnow et al., 2005; Han et al., 2007). CREB is also essential for ocular dominance plasticity, which is mediated by competitive interactions between bilateral monocular inputs (Mower et al., 2002). Acute expression of dominant-negative CREB in the primary visual cortex prevents ocular dominance plasticity, suggesting that the relative level of CREB function may underlie the competitive interaction responsible for its development (Pham et al., 1999; Mower et al., 2002).

A recent study has provided cell biological imaging evidence that LA neurons with increased CREB levels are preferentially recruited for inclusion into a fear memory trace (Han et al., 2007). Taking advantage of herpes simplex virus (HSV)-mediated gene transfer, this study applied a cellular imaging technique to visualize neurons activated during learning or memory retrieval. Auditory fear conditioning was used as a memory task, based on the fact that its circuitry is well described and because fear memory is rapidly acquired and stable, even with a single paring of a conditioned stimulus (CS)—a tone—and an unconditioned stimulus (US)—an aversive foot-shock (Davis, 1992; LeDoux, 2000; Fanselow and Gale, 2003; Maren and Quirk, 2004). On the basis of the previous observation that CREB regulates ocular dominance plasticity in the developing brain, which is mediated by competitive interaction between bilateral monocular inputs, and the critical role of CREB during memory formation, it was hypothesized that CREB might play an important role in the adult brain in determining which neurons are selected to encode memory. This idea was tested by investigating whether manipulation of CREB levels in a small subset of neurons in the lateral nucleus of the amygdala affected the neuronal selection process for fear memory. If fear memory is encoded and stored in sparsely distributed neurons in the LA, and CREB is a critical factor for the selection of these neurons during memory formation, the prediction is that CREB would be selectively activated in a small subset of neurons during fear memory training. To test this possibility, CREB activation was monitored by detecting the phosphorylation of endogenous CREB in the LA after fear conditioning training using an antibody against phosphorylated CREB. CREB was activated in about 20% of total LA neurons, and CREB activation was highly specific to the tone-shock paring condition, but not to other control conditions, such as tone or shock alone or home cage, suggesting that CREB was specifically activated in a small subset (~20%) of LA neurons by fear conditioning. If CREB is activated after fear conditioning, what is its functional role in fear memory formation? As noted above, transiently increasing CREB function in about 20% of LA neurons in rats enhanced fear memory in a fear potentiated startle-behavior paradigm (Josselyn et al., 2001). The role of CREB activation in a small subset of LA neurons in fear memory in mice was determined by increasing CREB levels in about 10–20% of LA neurons, after which these CREB overexpressing mice were trained with auditory fear conditioning. A disproportionate increase in CREB expression in only a subset of LA neurons was obtained by stereotaxic injection of HSV viral vectors (Carlezon et al., 2000; Neve et al., 2005) containing CREB fused with green fluorescent protein (HSV-CREB-GFP) into the LA of CREB^α δ−/−^ mice containing targeted deletions of CREBα and δ, the two main isoforms of CREB (Hummler et al., 1994; Pandey et al., 2000). Viral vectors expressing GFP alone (HSV-GFP) was used as controls. CREB^α δ−/−^ mice have been reported to exhibit memory impairment in auditory fear conditioning (Bourtchuladze et al., 1994; Frankland et al., 2004). Interestingly, when tested 1 day after auditory fear conditioning, CREB^α δ−/−^ mice injected with HSV-CREB-GFP showed a normal freezing response to the CS tone, whereas control mice injected with HSV-GFP showed no significant CS-evoked freezing, suggesting that expression of CREB in as few as 10–20% of LA neurons completely rescued impaired fear memory in CREB-deficient mice. Moreover, increasing CREB function in a subset of LA neurons in wild-type mice enhanced fear memory. One explanation for these behavioral results is that LA neurons injected with CREB are selectively recruited to encode memory. Alternately, although less likely, increasing CREB function in a small subset of LA neurons might enhance the general amygdala function to allow for more efficient memory encoding. To test if neurons injected with CREB are preferentially recruited into a fear memory trace, Josselyn and colleagues monitored activation of CREB-injected and non-injected neighboring neurons during fear-memory training or memory retrieval by molecular imaging of neuronal activity using the Arc catFISH technique. Because Arc mRNA, once induced by learning-related neuronal activity, stays in the nucleus for only 3–5 min after neuronal activity, the nuclear Arc mRNA signal can be used as a signature to provide precise temporal resolution of recently activated neurons (Guzowski et al., 2005). These imaging analyses provided evidence that CREB-injected LA neurons were preferentially activated during both fear conditioning and fear memory retrieval. An analysis of the number of neurons that were both CREB-positive and Arc-positive showed that CREB-injected neurons had about a 10-fold higher probability of being Arc-positive than non-injected neighboring neurons in CREB-deficient mice and about a 3-fold higher probability in wild-type mice Figure 1. If the functional level of CREB were a key factor for neuronal selection, then lowering CREB level would be expected to make neurons less likely to be selected for inclusion in the memory trace. This possibility was tested using CREB^S133A^, a dominant-negative CREB variant that cannot be phosphorylated at Ser133. Indeed, neurons injected with CREB^S133A^ were about 12-times less likely to be Arc-positive than non-infected neighboring neurons. Since CREB is a transcription factor, it is possible that Arc mRNA was induced in CREB-infected neurons not because these neurons were selectively activated during behavioral training or testing, but because CREB expression itself activated Arc mRNA transcription. This possibility was tested by injecting wild-type CREB or CREB^Y134F^, a constitutively active CREB, into the LA and monitoring Arc induction 3 days later by Arc *in situ* hybridization. Imaging data demonstrated that increasing CREB expression, in and of itself, was not sufficient to induce Arc mRNA. The Arc mRNA levels under these conditions were similar to baseline levels and, importantly, CREB-positive neurons were no more likely to be Arc-positive than their non-infected neurons, suggesting that Arc mRNA was induced in CREB-infected neurons because these neurons were preferentially activated during behavioral training or testing. Supporting this, it has also been shown in cell culture and *in vivo* that the phosphorylation status of CREB can be uncoupled from CREB-mediated gene expression (Chawla et al., 1998; Hardingham et al., 1999; Barrett et al., 2011). These results provide evidence that neurons in the LA are competitively selected for recruitment into a fear memory trace at the time of fear learning and show that the level of CREB function is one of the critical factors in determining this neuronal selection.

**Figure 1:**
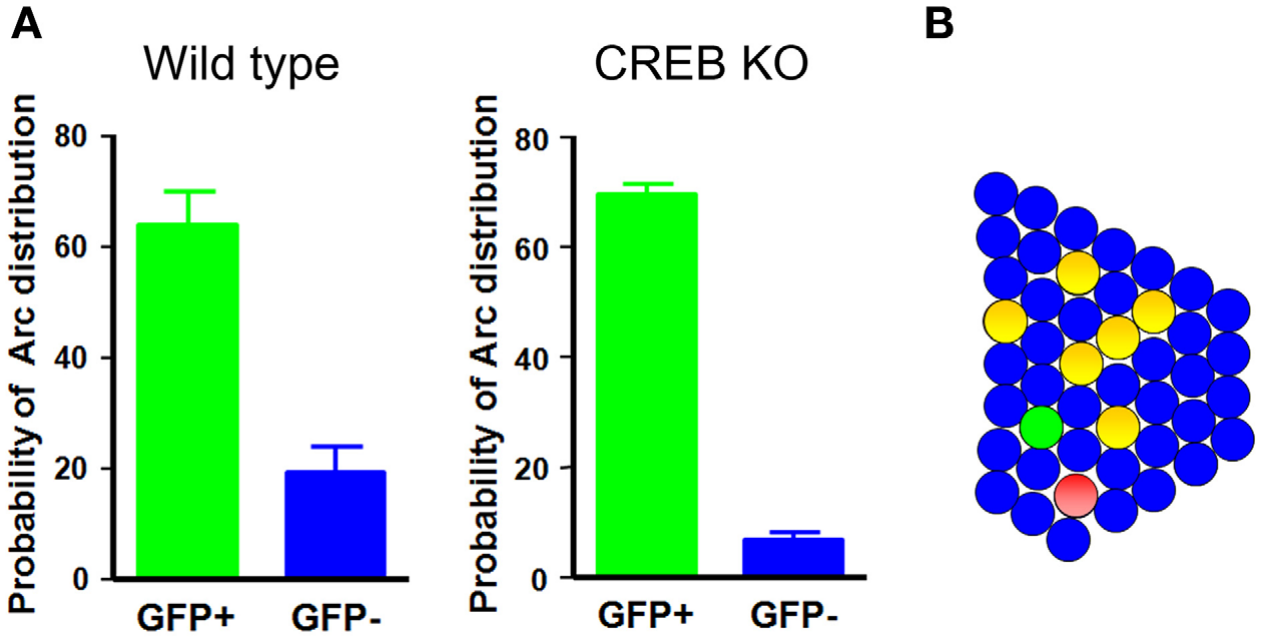
**Neurons with increased CREB function in the LA are preferentially recruited into the fear memory trace. (A)** In wild-type and CREB-deficient mice, neurons injected with CREB are about 3- and 10-times more likely, respectively, to be Arc-positive than their neighbors. **(B)** A schematic illustration of the LA network. Green circles, CREB-positive neurons; red circles, Arc-positive neurons; yellow circles, double-positive neurons.

However, this study did not directly prove that the neurons with higher levels of CREB are actually essential for fear memory storage or later expression. In a subsequent study, causal relationships were examined using the combined approach of inducible diphtheria toxin receptor (iDTR) transgenic mice and toxin-mediated cell ablation (Han et al., 2009). The rationale for these experiments is that, if the neurons with higher levels of CREB are essential for fear memory, then post-training ablation of these specific neurons should disrupt established fear memory. In the iDTR mouse, the expression of a simian DTR transgene is activated by Cre-recombinase-mediated cleavage of a stop cassette located in the upstream region of the DTR transgene, thereby inhibiting expression of the DTR gene. These neurons become sensitive to diphtheria toxin (DT)-induced apoptotic cell death only in the presence of Cre-recombinase (Yamaizumi et al., 1978; Buch et al., 2005). Thus, this technique allows for target-specific cell ablation in a temporally and spatially restricted manner. Selectively targeting and ablation of neurons injected with CREB was accomplished by introducing an HSV-CREB-IRES-Cre (CREB-Cre) construct, or HSV-GFP-IRES-Cre (GFP-Cre) control, into the LA of iDTR mice. In these HSV constructs, the HSV virus immediate-early gene promoter IE4/5 was used to drive the expression of CREB and an IRES sequence was used to drive the expression of Cre-recombinase. In the first experiment, Arc mRNA imaging confirmed that the neurons injected with CREB-Cre were also preferentially activated during fear memory retrieval, as was the case with neurons injected with CREB. Next, experiments were performed to determine whether systemic administration of DT in iDTR mice specifically ablated target neurons injected with HSV virus. Groups of mice were bilaterally injected with CREB-Cre or GFP-Cre into the LA, and 3 days later DT was delivered by intraperitoneal injection. Cell ablation was monitored using two markers of apoptotic cell death—activated caspase 3 and terminal deoxynucleotidyl transferase dUTP nick end-labeling (TUNEL)—and quantified by counting the number of activated caspase 3-positive or TUNEL-positive LA neurons. The results revealed specific cell ablation in both CREB-Cre- and GFP-Cre-injected iDTR mice only at the targeted LA regions where HSV virus was injected, demonstrating specific cell ablation of neurons injected with HSV-CREB-Cre or HSV-GFP-Cre in the iDTR/DT system. What effect would this cell ablation have on the established memory? This important question was tested by injecting iDTR mice with CREB-Cre or GFP-Cre and training them with auditory fear conditioning 3 days after HSV virus injection. DT was injected immediately after the initial test of auditory fear memory. When re-tested 2 days after DT injection, CREB-Cre mice, but not GFP-Cre mice, displayed a low level of freezing upon CS tone presentation, indicating that fear memory was disrupted by the specific deletion of CREB-overexpressing LA neurons. Notably, simply removing fewer than 15% of LA neurons did not impair fear memory, a result consistent with a previous report (Rumpel et al., 2005). Repeated tests over several days (2, 5, and 12 days after DT injection) showed a persistently low level of CS-induced freezing in CREB-Cre mice. Moreover, there was no sign of fear memory renewal or spontaneous recovery. A series of control experiments demonstrated that the disruption of fear memory by post-training ablation of neurons overexpressing CREB was highly specific. First, memory disruption was not due to non-specific interference of general amygdala circuit functions. CREB-overexpressing neurons were first deleted by DT injection, and then the mice were trained and tested. Under these conditions, every mouse displayed normal auditory fear memory formation. Second, pre-existing memory (i.e., before CREB manipulation) was spared after cell ablation. Third, CREB-Cre mice could re-learn auditory fear conditioning even after cell ablation, suggesting that memory disruption was not due to the impairment of general amygdala memory function. Fourth, although it is possible that CREB overexpression could non-specifically alter amygdala memory circuits such that the fear memories became more susceptible to the ablation of small numbers of CREB-expressing neurons within the network, this was not the case because the ablation of GFP-Cre neurons with a CREB-overexpression background did not disrupt established fear memory. These findings may be the first to show disruption of a specific memory by ablating selective neurons within a distributed network.

The finding that LA neurons with a higher level of CREB are essential for the recall of fear memory was reconfirmed and extended by another study using a reversible inactivation strategy (Zhou et al., 2009). In this study, the *Drosophila* allatostatin receptor (AlstR), which activates the GIRK (G protein-gated inwardly-rectifying) type of K^+^ channel upon binding of a selective ligand (Birgül et al., 1999), was used to decrease the excitability of CREB-overexpressing target neurons within a specific time window. This study also used HSV virus-mediated gene transfer to deliver CREB and AlstR genes into a subset of LA neurons. Inactivating CREB neurons at the time of auditory fear memory test produced substantial and reversible memory impairment. Furthermore, the same manipulations disrupted expression of the memory for conditioned taste aversion, another type of amygdala-dependent memory. This study also provided electrophysiological evidence supporting the idea that CREB increases the intrinsic excitability of LA neurons. CREB overexpression in LA neurons did not change resting membrane potential, input resistance, spike amplitude, or spike half-width, but it significantly lowered the threshold for action potential firing. CREB has been reported to increase neuronal excitability in different brain regions (Dong et al., 2006; Lopez de Armentia et al., 2007; Viosca et al., 2009). Dong and colleagues found that CREB increases the excitability of medium spiny neurons in the nucleus accumbens (Dong et al., 2006). Expression of active CREB in these neurons increased excitability, whereas downregulation of CREB by expressing dominant-negative CREB decreased excitability. The electrophysiological recordings from transgenic mice expressing a constitutively active form of CREB, VP16-CREB, showed that enhancing CREB activity increased the intrinsic excitability of hippocampal CA1 and basal amygdala pyramidal neurons (Lopez de Armentia et al., 2007; Viosca et al., 2009). Consistent with the observed CREB-induced increase in excitability, CREB function has also been reported to stimulate the expression of the voltage-dependent Na^+^ channel 1β subunit and inhibit the expression of the voltage-dependent K^+^ channel K_V_1.4 subunit (McClung and Nestler, 2003). The regulation of neuronal excitability can be a potential molecular mechanism underlying CREB-dependent neuronal selection, as suggested previously (Won and Silva, 2008; Silva et al., 2009; Zhou et al., 2009; Benito and Barco, 2010; Josselyn, 2010). Learning-induced changes in neuronal excitability have also been reported. Electrophysiological recording from rabbit CA1 pyramidal neurons in slices prepared after acquisition of trace eyeblink conditioning increased neuronal excitability observed as early as 1 h after the learning (Moyer et al., 1996). Increased neuronal excitability was observed in rat piriform cortex after operant conditioning and hippocampal CA1 pyramidal neurons after olfactory discrimination learning (Saar et al., 1998; Zelcer et al., 2006). Notably, a recent study reports that intrinsic neuronal excitability in basolateral amygdala (BLA) pyramidal neurons is differentially modified by positive and negative olfactory learning. Thus, neuronal excitability in BLA neurons was decreased after odor fear conditioning (Motanis et al., 2012). However, whether auditory fear conditioning actually induces the changes in excitability in a subpopulation of LA neurons and, if so, whether these changes are correlated with CREB activation *in vivo*, have not yet been demonstrated. An alternative mechanism for neuronal selection by CREB has been proposed based on the previous observation that CREB regulates the density or morphology of dendritic spines. Structural and electrophysiological analyses of rat hippocampal CA1 neurons have revealed that virally delivered, constitutively active CREB generates silent synapses (Marie et al., 2005). In contrast, dominant-negative CREB decreases spine head size in visual cortex neurons (Suzuki et al., 2007). Both increased excitability and generation of more plastic synapses by CREB could increase the probability of neurons with higher CREB levels being activated during fear memory formation and the subsequent consolidation process. Taken together, these recent findings provide compelling evidence in support of the hypothesis that CREB is a key factor governing the selection of neurons for inclusion into the fear memory trace in the amygdala and shed light on how a memory engram is formed within a complex neural circuit.

These recent findings leave a number of important unanswered questions that can be addressed in future studies. For instance, the amygdala consists of heterogeneous cell populations, raising the question of exactly what types of cells participate in the fear memory engram and how they function. In addition, the studies described above artificially manipulated the level of CREB function in a subset of neurons to forcibly bias CREB levels in amygdala circuit. However, it remains unclear whether different cells in amygdala network actually have different levels of endogenous CREB function at any particular moment; if so, how are they generated and coordinated? A real-time reporter system capable of reflecting the functional level of endogenous CREB in a defined cell population would help address this question. Although monitoring CREB activation immunohistochemically using an antibody against the phosphorylated form of CREB has demonstrated that endogenous CREB is activated in about 20% of LA neurons after auditory fear conditioning, how the endogenous CREB is activated in those specific neurons and, more importantly, how the size of the memory engram is determined within a given neural circuit, remain unknown. In a natural setting, animals experience a virtually continuous flow of information. Thus, the more natural situation is probably that the history of previous neuronal activity affects the level of functional CREB in each individual neuron. Accordingly, it is very likely that the prior activation of CREB would affect subsequent information-coding processes coming within a limited time window—a metaplasticity-like mechanism. Interestingly, recent a study using a rat fear-potentiated startle paradigm reported evidence for metaplasticity-like regulation of fear memory. In this study, rats trained with a relatively weak single pairing of a light with shock, conditions that are insufficient for either short- or long-term fear memory, primed upcoming learning such that another trial given within a limited time window lasting from ~60 min to 3 days resulted in the formation of a long-lasting and robust fear memory (Parsons and Davis, 2012). These results suggest that the network state at the time of learning can influence new information encoding and storage. Interestingly, single pairing in this condition induced phosphorylation of CREB in amygdala neurons. Given the importance of CREB levels for selection of neurons for inclusion into the memory trace, CREB activation by prior neuronal activity would also be expected to affect the memory-allocation process. An intriguing question in this context is whether one neuron can take part in multiple memory traces. Related with this issue, CREB-dependent neuronal selection model implies that there should be a mechanism by which the functional level of CREB is precisely regulated to allow it returning to the basal state from activation; otherwise same neurons will become part of all the newly acquired memory traces. As previously suggested (Won and Silva, 2008; Benito and Barco, 2010), transcriptional negative feedback mechanisms might be important for this regulation.

To date, most studies, including those reviewed here, have used a perturbation strategy to establish a causal link between particular sets of neurons and a memory engram. However, another key issue in identifying a memory engram is to determine if activating specific sets of neurons is sufficient to induce recall of memory. A recently published study using an optogenetic technique reported that reactivation of dentate gyrus (DG) neurons that are active during contextual fear memory training (i.e., seemingly those that represent context information associated with aversive foot-shock during conditioning) is sufficient to drive the recall of that memory (Liu et al., 2012). One possible scenario in this case is that activation of the tagged DG neurons may, in turn, activate the amygdala memory engram neurons that store the information for the association of the context CS and US, which then drives the associated fear behavioral response. A study of the amygdala circuitry that combined genetic tagging of *c-fos*-active neurons and immediate-early gene imaging demonstrated that neurons in the BLA that are activated during fear conditioning are reactivated during fear memory recall, and further showed that the number of reactivated neurons is positively correlated with the behavioral expression of the fear memory (Reijmers et al., 2007). This report suggests that reactivation of amygdala neurons active during fear memory training may induce recall of fear memory. However, whether directly activating a specific subset of neurons in the amygdala can induce fear memory recall remains unknown. With new advances in techniques that allow scientists to control the firing of defined cell populations (Boyden et al., 2005; Shapiro et al., 2012; Tye and Deisseroth, 2012), it will be possible to test whether those LA neurons with a relatively higher level of CREB function are sufficient for fear memory recall. Another fascinating issue arising from current views on the dynamic nature of the memory trace (Dudai, 2012) is the effect of this direct reactivation of a fear memory trace, which is sufficient to induce behavioral recall of memory, on the memory trace itself. It is well established that retrieval of fear memory can induce a reconsolidation process (Misanin et al., 1968; Nader et al., 2000) and repeated presentation of CS without US induces extinction (Myers and Davis, 2007). Whether reactivation of an established fear memory trace causes reconsolidation or extinction-like changes remains an unanswered question. It has been thought that subpopulations of neurons that are active during behavior training may be reactivated later in the brain, and this activity replay may cause strengthening of a previously acquired memory (Bunch and Magdisck, 1933; Wilson and McNaughton, 1994). Thus, it will be interesting to determine whether repeated reactivation of neurons injected with CREB enhances the established fear memory.

The studies reviewed here represent the first steps toward the ultimate goal of completely understanding how and where new information is encoded and stored by an ensemble of neurons in a complex neural circuit. This research will also provide insights into the mechanisms underlying learning and memory defects in various human mental disorders and neurodegenerative diseases.

## Conflict of interest statement

The authors declare that the research was conducted in the absence of any commercial or financial relationships that could be construed as a potential conflict of interest.
